# Supplementary material for: Predicting the Structure of Enzymes with Metal Cofactors: The Example of [FeFe] Hydrogenases
Source: Int J Mol Sci. 2024 Mar 25;25(7):3663. doi: 10.3390/ijms25073663 (PMC11011570; doi:10.3390/ijms25073663)
Supplement: Supplementary file 1 [file ijms-25-03663-s001.zip › ijms-2857370-SM/Blast_Clustal_Alignments/Clustal-Omega.rtf]

CLUSTAL O(1.2.4) multiple sequence alignmentKAI3438965.1                     ------------------------------------------------------------	0C.Variabilis                     MEALVRRGLQSPDQALRLLGVARAICVGLSRSLPALAASAEQFAEQYPKLKLKVNGREVT	60C.r_HydA1                        ------------------------------------------------------------	0Tetradesmus                      ------------------------------------------------------------	0C.sp-DT                          ------------------------------------------------------------	0C.fusca                          ------------------------------------------------------------	0Cl.Pasterianumn                  ------------------------------------------------MKTIIINGVQFN	12Desulfovibrio_desulfuricans      ------------------------------------------------------------	0                                                                                             KAI3438965.1                     ----------------MFSGAVKIGDLHDFISPAQACVVNLEGGKLPNNVL---------	35C.Variabilis                     VPEGTSVLNACREAGAYVPTLCTHPRLPTTPGTCRICMVETGGGQLKPACATPAWEGMEV	120C.r_HydA1                        ------------------------------------------------------------	0Tetradesmus                      ------------------------------------------------------------	0C.sp-DT                          ------------------------------------------------------------	0C.fusca                          ------------------------------------------------------------	0Cl.Pasterianumn                  TDEDTTILKFARDNNIDISALCFLNNCNNDINKCEICTVEVEGTGLVTACDTLIEDGMII	72Desulfovibrio_desulfuricans      ------------------------------------------------------------	0                                                                                             KAI3438965.1                     ------------------------------------------------------------	35C.Variabilis                     QTATDKVQESIRGVLSLMKANHPSDCMNCDASGRCEFQDLISRYNVKDVLPKLKTYSHEW	180C.r_HydA1                        ------------------------------------------------------------	0Tetradesmus                      ------------------------------------------------------------	0C.sp-DT                          ------------------------------------------------------------	0C.fusca                          ------------------------------------------------------------	0Cl.Pasterianumn                  NTNSDAVNEKIKSRISQLLDIHEFKCGPCNRRENCEFLKLVIKYKARASKPFLPKD----	128Desulfovibrio_desulfuricans      ---------------------------------MSRTVMERIEYEMHTPDPKADPD----	23                                                                                             KAI3438965.1                     -------------------------------ADAEAGTVQLQPRKPQGFAQT-LPAAADE	63C.Variabilis                     DAEVQADFEHFHDSSSTALTLDLEKCIKCGRCVTMCGQVQQMNVLGMINRSRMAHPGVLI	240C.r_HydA1                        -----------------MSALVLKPCAAVSIRGSSCRARQVAPRAPL-AASTVRVALATL	42Tetradesmus                      -----------------MPEW--QPGGR---YAVSV--------RPP-VNRRAVVAAERR	29C.sp-DT                          ----------------------------------MC--------CPV-VAS--RHAGRAR	15C.fusca                          ----------------------------------MC--------CPV-VAS--RHAGRAR	15Cl.Pasterianumn                  -------KTEYVDERSKSLTVDRTKCLLCGRCVNACGKNTETYAMKFLNKNGKTIIGAED	181Desulfovibrio_desulfuricans      --------------KLHFVQIDEAKCIGCDTCSQYCPTAAIFGEM------G------EP	57                                                                                             KAI3438965.1                     AIKVSLHDCLACSGCVTSAEAV-LLQHQSA----GELLQRLADP-DWTVVVSLSPQSIAA	117C.Variabilis                     EEALDHSKCIECGQCSSVCPVGAIVEHSEWRQVLDALENK-----QKVMVVQTAPSVRVS	295C.r_HydA1                        EAPARRLGN----VACAAAAPAAEAPLSHVQQALAELAKPKDDPTRKHVCVQVAPAVRVA	98Tetradesmus                      RLVVRAAGP----TAECDCPPAPAPKAPHWQQTLDELAKPKEQ--RKVMIAQIAPAVRVA	83C.sp-DT                          HVAVRAAGP----TSECDCPPTPQAKLPHWQQALDELAKPKES--RRLMIAQIAPAVRVA	69C.fusca                          HVAVRAAGP----TSECDCPPTPQAKLPHWQQALDELAKPKES--RRLMIAQIASAVRVA	69Cl.Pasterianumn                  EKCFDDTNCLLCGQCIIACPVAALSEKS-HMDRVKN---ALNA-PEKHVIVAMAPSVRAS	236Desulfovibrio_desulfuricans      HSIPHIEACINCGQCLTHCPENAIYEAQSWVPEVEK---KLKD-GKVKCIAMPAPAVRYA	113                                               .   .                               .  :     :KAI3438965.1                     LAAVHRLS-SAECAARLTAFLRHLGVAAVFDIAAARQVALAEAAREFLQRYRSSRGGSTA	176C.Variabilis                     IGEELGLAPGTVETGQMVAAQRALGFDYVFDSDFSADLTIMEEGTELLQRLGAAWRAETA	355C.r_HydA1                        IAETLGLAPGATTPKQLAEGLRRLGFDEVFDTLFGADLTIMEEGSELLHRLTEHL-----	153Tetradesmus                      IAETMGLNPGDVTVGQMVTGLRMLGFDYVFDTLFGADLTIMEEGTELRHRLQDHL-----	138C.sp-DT                          IAETIGLAPGDVTIGQLVTGLRMLGFDYVFDTLFGADLTIMEEGTELLHRLQDHL-----	124C.fusca                          IAETIGLAPGDVTIGQLVTGLRMLGFDYVFDTLFGADLTIMEEGTELLHRLQDHL-----	124Cl.Pasterianumn                  IGELFNMGFGVDVTGKIYTALRQLGFDKIFDINFGADMTIMEEATELVQRIEN-------	289Desulfovibrio_desulfuricans      LGDAFGMPVGSVTTGKMLAALQKLGFAHCWDTEFTADVTIWEEGSEFVERLTKK------	167                                 :.    :  .     ::    : **.   :*     :::: * . *: .*          KAI3438965.1                     EAAAAAGQDAAGGGDDMDVDRPKPSSDGHRNAVGGGSPGPLPMLASACPGWVCYAEKTHG	236C.Variabilis                     AQDAAAGSWAAAK------------QGHGEGEAHGHAPGPLPMFTSCCPAWINLVEKSYP	403C.r_HydA1                        -------------------------------EAHPHSDEPLPMFTSCCPGWIAMLEKSYP	182Tetradesmus                      -------------------------------EQHPNKEEPLPMFTSCCPGWVAMVEKSNP	167C.sp-DT                          -------------------------------EQHPNKEEPLPMFTSCCPGWVAMVEKSNP	153C.fusca                          -------------------------------EQHPNKEEPLPMFTSCCPGWVAMVEKSNP	153Cl.Pasterianumn                  -------------------------------------NGPFPMFTSCCPGWVRQAENYYP	312Desulfovibrio_desulfuricans      ------------------------------------SDMPLPQFTSCCPGWQKYAETYYP	191                                                                        *:* ::*.**.*    *.   KAI3438965.1                     DHVLPYIATGKSPQAVLGTLVKRQWCAAAGLAPDRVYHCSVMPCYDKKLEGARDDFWLPG	296C.Variabilis                     -ELIPHLSSCKSPQMMMGAVVKHYWAKKKGLKPEDVCLVGIMPCTAKKHETERKEFRNE-	461C.r_HydA1                        -DLIPYVSSCKSPQMMLAAMVKSYLAEKKGIAPKDMVMVSIMPCTRKQSEADRDWFCVDA	241Tetradesmus                      -ELIPYLSSCKSPQMMLGAVIKNYFAAEAGAKPEDICNVSVMPCVRKQGEADREWFNTTG	226C.sp-DT                          -ELIPYLSSCKSPQMMLGAVIKNYYAQQVGVQPSDICNVSVMPCVRKQGEADREWFNTT-	211C.fusca                          -ELIPYLSSCKSPQMMLGAVIKNYYAQQVGVQPSDICNVSVMPCVRKQGEADREWFNTTG	212Cl.Pasterianumn                  -ELLNNLSSAKSPQQIFGTASKTYYPSISGLDPKNVFTVTVMPCTSKKFEADRPQMEKD-	370Desulfovibrio_desulfuricans      -ELLPHFSTCKSPIGMNGALAKTYGAERMKYDPKQVYTVSIMPCIAKKYEGLRPELKSS-	249                                  .::  .:: ***  : .:  *          *. :    :***  *: *  *  :    KAI3438965.1                     T-SVPETDCVLATTELQELLEQRQADLHSLQGIPFDAILPAAAAAAAAPSGTRASSSGGS	355C.Variabilis                     -NGAYDCDYVITTREFGHMLRHKKIPMPSLKPEEFDNPLGEATGAAALF-----------	509C.r_HydA1                        DPTLRQLDHVITTVELGNIFKERGINLAELPEGEWDNPMGVGSGAGVLF-----------	290Tetradesmus                      AGG-ANVDHVMTTAELGKIFVERGIKLNDLQESPFDNPVGEGSGGGVLF-----------	274C.sp-DT                          -GLARDVDHVVTTAEVGKIFLERGIKLNELPESNFDNPIGEGTGGALLF-----------	259C.fusca                          AGLARDVDHVVTTAEVGKIFLERGIKLNELPESNFDNPIGEGTGGALLF-----------	261Cl.Pasterianumn                  --GLRDIDAVITTRELAKMIKDAKIPFAKLEDSEADPAMGEYSGAGAIF-----------	417Desulfovibrio_desulfuricans      --GMRDIDATLTTRELAYMIKKAGIDFAKLPDGKRDSLMGESTGGATIF-----------	296                                      : * .::* *.  :: .    : .*     *  :   :...              KAI3438965.1                     SAQHDTVQLPTSSSSGSGSGVTATASSGSGGYLEHVFRSAARELFGL-------------	402C.Variabilis                     --------------------------GATGGVMEAAIRTAYEIAAGEPLPKLEVEAVRGV	543C.r_HydA1                        --------------------------GTTGGVMEAALRTAYELFTGTPLPRLSLSEVRGM	324Tetradesmus                      --------------------------GTTGGVMEAALRTVYEVVTQKPLDRIVFEDVRGL	308C.sp-DT                          --------------------------GTTGGVMEAALRTVYEVVTQKPMGRVDFEEVRGL	293C.fusca                          --------------------------GTTGGVMEAALRTVYEVVTQKPMGRVDFEEVRGL	295Cl.Pasterianumn                  --------------------------GATGGVMEAALRSAKDFAENAELEDIEYKQVRGL	451Desulfovibrio_desulfuricans      --------------------------GVTGGVMEAALRFAYEAVTGKKPDSWDFKAVRGL	330                                                           . :** :* .:* .                    KAI3438965.1                     --------ELP--------------------------PGPLPVKVGRNADLRELSLEAPD	428C.Variabilis                     KGVKEATLTLPANDTTL--------------------------------------KAGVA	565C.r_HydA1                        DGIKETNITMVPAPGSKFEELLKHRAAARAEAAAHGTPGPLAWDGGA----GFTSEDGRG	380Tetradesmus                      EGIKESTLHLTPGPTSPFKAFAG--------------------------------A-DGT	335C.sp-DT                          EGIKEAEITLKPGDDSPFKAFAG--------------------------------A-DGQ	320C.fusca                          EGIKEAEITLKPGDDSPFKAFAG--------------------------------A-DGQ	322Cl.Pasterianumn                  NGIKEAEVE-------------------------------------------------IN	462Desulfovibrio_desulfuricans      DGIKEATVN-------------------------------------------------VG	341                                                                                             KAI3438965.1                     GTVLRFAAAYGFRNIQGLMRKVKLGRCEYDYVEVMACPSGCLNGGGQPKPTAKQTPAQ--	486C.Variabilis                     GKEIRVAVASGIGNARHLLQRIQAGEAHYDFVEVMACPGGCIGGGGQPKTHDP-----DA	620C.r_HydA1                        GITLRVAVANGLGNAKKLITKMQAGEAKYDFVEIMACPAGCVGGGGQPRSTDK-----AI	435Tetradesmus                      GITLNIAVANGLGNAKKLIKQLAAGESKYDFIEVMACPGGCIGGGGQPRSADK-----QI	390C.sp-DT                          GITLKIAVANGLGNAKKLIKSLSEGKAKYDFIEVMACPGGCIGGGGQPRSTDK-----QI	375C.fusca                          GITLKIAVANGLGNAKKLIKSLSEGKAKYDFIEVMACPGGCIGGGGQPRSTDK-----QI	377Cl.Pasterianumn                  NNKYNVAVINGASNLFKFMKSGMINEKQYHFIEVMACHGGCVNGGGQPHVNPKDLEKVDI	522Desulfovibrio_desulfuricans      GTDVKVAVVHGAKRFKQVCDDVKAGKSPYHFIEYMACPGGCVCGGGQPVMP-GVLEAMDR	400                                 .   ..*.  *  .   .      ..  *.::* *** .**: *****            KAI3438965.1                     LLEQLEELYAAG---GIAARPEADPALQQLYMSFVQGEPGSQAAHQLMHTHYHKRDKTVT	543C.Variabilis                     VLKRMGAIYQVD-KSLALRKSHENPSIHKIYAEFL-GQPGGELSHKLLHTHYTDHSVDTL	678C.r_HydA1                        TQKRQAALYNLD-EKSTLRRSHENPSIRELYDTYL-GEPLGHKAHELLHTHYVAGGVEEK	493Tetradesmus                      LQKRQAAMYDLD-ERAVIRRSHENPLIGALYEKFL-GEPNGHKAHELLHTHYVAGGVPDE	448C.sp-DT                          LQKRQQAMYNLD-ERSAIRRSHENPFIQALYDKFL-GAPNSHKAHDLLHTHYVAGGIPEE	433C.fusca                          LQKRQQAMYNLD-ERSTIRRSHDNPFIQALYDKFL-GAPNSHKAHDLLHTHYVAGGIPEE	435Cl.Pasterianumn                  KKVRASVLYNQD-EHLSKRKSHENTALVKMYQNYF-GKPGEGRAHEILHFKYKK------	574Desulfovibrio_desulfuricans      TTTR---LYAGLKKRLAMASA--NKA----------------------------------	421                                    :   :*              :                                    KAI3438965.1                     ATLADW---------------------------------	549C.Variabilis                     PSVRELGGSGEVAKRAALTAAGEMRYKRIAMVGDPSAKR	717C.r_HydA1                        DEKK-----------------------------------	497Tetradesmus                      K--------------------------------------	449C.sp-DT                          K--------------------------------------	434C.fusca                          K--------------------------------------	436Cl.Pasterianumn                  ---------------------------------------	574Desulfovibrio_desulfuricans      ---------------------------------------	421##################################################################################################################################################################################################################################################################  Percent Identity  Matrix - created by Clustal2.1 ##     1: KAI3438965.1                 100.00   28.36   30.73   28.61   28.72   28.57   26.18   27.87     2: C.Variabilis                  28.36  100.00   46.27   47.03   48.94   48.24   39.65   39.42     3: C.r_HydA1                     30.73   46.27  100.00   59.91   62.44   61.93   38.26   37.14     4: Tetradesmus                   28.61   47.03   59.91  100.00   81.52   81.15   38.20   38.11     5: C.sp-DT                       28.72   48.94   62.44   81.52  100.00   99.31   38.94   40.50     6: C.fusca                       28.57   48.24   61.93   81.15   99.31  100.00   38.44   39.94     7: Cl.Pasterianumn               26.18   39.65   38.26   38.20   38.94   38.44  100.00   41.97     8: Desulfovibrio_desulfuricans   27.87   39.42   37.14   38.11   40.50   39.94   41.97  100.00                                                                        
