# Supplementary material for: Predicting the Structure of Enzymes with Metal Cofactors: The Example of [FeFe] Hydrogenases
Source: Int J Mol Sci. 2024 Mar 25;25(7):3663. doi: 10.3390/ijms25073663 (PMC11011570; doi:10.3390/ijms25073663)
Supplement: Supplementary file 1 [file ijms-25-03663-s001.zip › ijms-2857370-SM/SM-tex/SM.pdf]

International Journal of
Molecular Sciences

MDPI

Article

Supplementary Materials—Predicting the Structure of Enzymes with Metal Cofactors: The Example of [FeFe] Hydrogenases

Simone Botticelli [1,2], Giovanni La Penna [2,3,*], Velia Minicozzi [1,2], Francesco Stellato [1,2], Silvia Morante [1,2], Giancarlo Rossi [1,2,4] and Cecilia Faraloni [5]

1 Department of Physics, University of Roma Tor Vergata, 00133 Rome, Italy
2 Section of Roma Tor Vergata, National Institute of Nuclear Physics, 00133 Rome, Italy
3 Institute of Chemistry of Organometallic Compounds, National Research Council, 50019 Florence, Italy
4 Museo Storico della Fisica e Centro Studi e Ricerche E. Fermi, 00184 Rome, Italy
5 Institute of Bioeconomy, National Research Council, 50019 Florence, Italy
* Correspondence: giovanni.lapenna@cnr.it

check for updates

Citation: Botticelli, S.; La Penna, G.; Minicozzi, V.; Stellato, F.; Morante, S.; Rossi, G.C.; Faraloni, C. . *Int. J. Mol. Sci.* **2024**, *1*, 0. https://doi.org/

Academic Editor: Ivo Crnolatac

Received: 19 January 2024
Revised: 12 March 2024
Accepted: 18 March 2024
Published: 25 March 2024

Copyright: © 2024 by the authors. Licensee MDPI, Basel, Switzerland. This article is an open access article distributed under the terms and conditions of the Creative Commons Attribution (CC BY) license (https://creativecommons.org/licenses/by/4.0/).

"Supplementary Materials" directory contains information to reproduce the work and results. This document helps in browsing these data and contains information not displayed in the main text.

1. Alignment and gene annotation

1. microalgae_info.xlsx file;
2. Blast&Clustal_Alignments directory;
3. AF_alignments directory;

microalgae_info file:
we have summarized available information about microalgae and cyanobacteria found in literature and relevant to this work.

Blast_Clustal_Alignments:
This directory contains four files and two sub-directories. The four files are:

- sequences.txt, a file with the 7 [FeFe] hydrogenase sequences used to perform all the alignments;
- BL90_NEW_Alignment.txt, a file with alignments results, using BLOSUM90 matrix, between KAI3438965.1 and benchmark sequences;
- PAM30-NEW-Alignment.txt, a file with alignments results, using PAM30 matrix, between KAI3438965.1 and benchmark sequences;
- Clustal-Omega.rtf, a file with multiple alignments Clustal-Omega algorithm.

The two sub-directories are:
Chlorella_Vulg211/11P_Nuclear_Protein_Blosum90_Alignment;
Chlorella_Vulg211/11P_Nuclear_Protein_Pam30_Alignment.

These sub-directories contain all alignments between the whole genome of Chlorella Vulgaris 211/11P and the 7 benchmark [FeFe] hydrogenases, respectively with BLOSUM90 and PAM30 matrix.

AF_alignments:
This directory contains a file, AlphaFoldREADME.txt, with the version and the parameters used to perform AlphaFold prediction calculations. The "msas" sub-directory contains AlphaFold alignments results. It is important to notice that AlphaFold results depend on the date of execution, because the information that is elaborated increases with time.

The date of the elaborated data-base is indicated in the execution line contained in the AlphaFoldREADME.txt file.

2. Structure prediction

Table S1. Residues belonging to the H-domain, aligned along with the *Cp*, *Dd*, *Cr*, and *Cvu* Hyd sequences. The secondary motifs assigned to the scaffold are h1-11 (11 α-helices) and b1-7 (7 β-strands forming 2 β-sheets).

Secondary motif	Species			
	CpI	*Dd* Hyd	*Cr* Hyd	*Cvu* Hyd
h1	212-219	89-96	33-40	93-100
h2	250-260	127-137	72-82	130-140
h3	268-287	145-164	90-109	148-167
h4	301-310	180-189	131-140	225-234
h5	324-340	203-219	154-170	249-265
h6	357-362	236-241	187-192	282-287
h7	381-390	260-269	215-224	308-317
h8	412-413	291-292	245-246	373-374
h9	414-415	293-294	247-248	378-379
h10	422-436	301-315	255-269	386-400
h11	476-484	356-364	355-363	443-451
b1	224-229	101-106	46-51	105-110
b2	264-267	141-144	86-89	144-147
b3	347-353	226-232	177-183	272-278
b4	377-380	256-259	210-213	304-307
b5	454-460	333-339	287-293	419-425
b6	465-472	344-351	343-350	431-438
b7	493-497	372-376	371-375	459-463

Initial structures of the three models studied in this work, as generated by AlphaFold (model **1**, left), SwissModel (model **2**, middle) and by the manual construction (see text, model **3**, right panel).

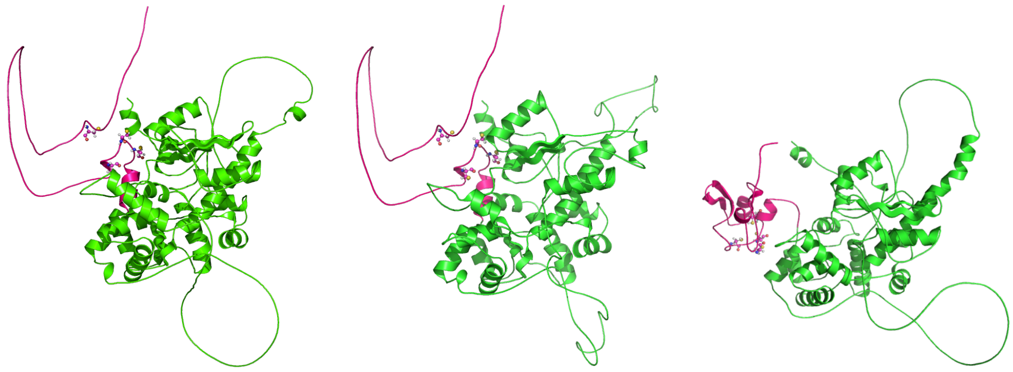

Figure S1. *Cvu* Hyd AlphaFold predicted model (left); SwissModel prediction (middle); manual construction (right). H- and F-domains are in green and purple colors, respectively, using residues specified in Table 1 of main text. Cys 21-72-75-78 are shown in ball and sticks. FeS clusters are not yet added to models.

Below the figures displaying the predicted aligned error (PAE) and the predicted accuracy index for structural prediction (plDDT) determined by AlphaFold for the highest-score structure.

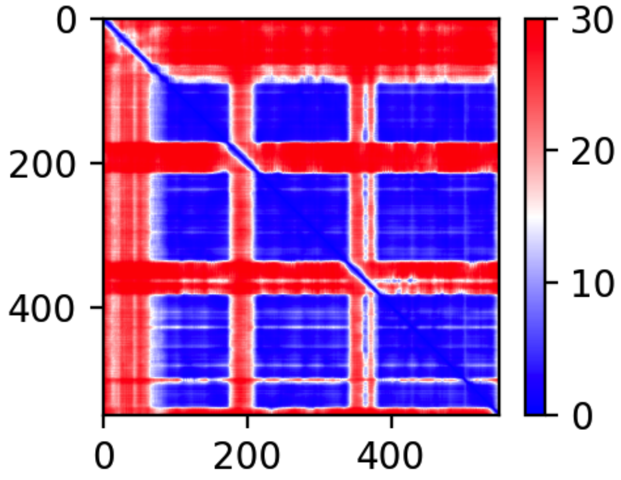

Figure S2. Predicted aligned error (PAE, in Å, see main text) as a function of the residue numbers for the AlphaFold structural prediction with highest score.

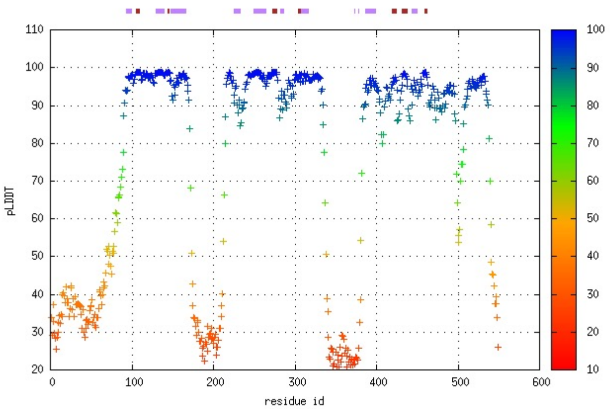

Figure S3. Index plDDT (see Methods in main text) as a function of the residue number for the AlphaFold structural prediction with highest score. Horizontal bars (top) display the range of secondary domains common to all available crystal structures (DSSP algorithm [1] used by AlphaFold, see also Figure S1): α-helices (purple); β-strands (brown).

3. Structure refinement

Force_Field directory contains the parameters used to compute forces on atoms of the FeS clusters and some commands to build initial configurations. The “topology_parameters” sub-directory contains the Charmm 36 force-field with the additional files required by FeS clusters adapted from Ref. [2]. The file gen.tcl can be used to build any initial model with no solution environment. The file gen.vmd performs the same task using VMD [3]. The preparation tools of NAMD [4], sometimes included in VMD, must be used. The inclusion of water and of NaCl salt must be performed with “solvate” and “autoionize” tools included in VMD. Simulation cell parameters are described in Mehods section of main text. Conversion.vmd file is used to generate the topology file for GROMACS input, as described in Ref. [5] via the VMD program.

The configurations obtained from those displayed in Figure S1, after energy minimizations are displayed in Figure S4 the three models (**1**, **2**, and **3**) are displayed with all the bonded FeS clusters (left, middle, and right panels, respectively).

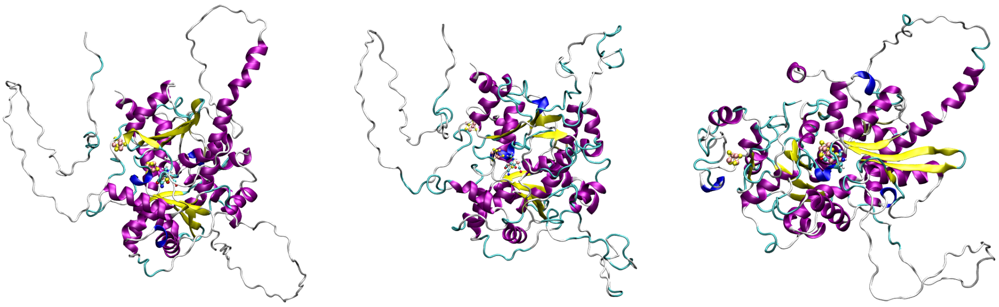

Figure S4. Protein models with the FeS clusters inserted and after energy minimization. AlphaFold model **1** (left); SwissModel model **2** (middle); "manual" construction **3** (right). Secondary domains are represented with STRIDE via VMD [3]: α-helices (purple); β-strands (yellow); 1-3 helix (blue); unstructured regions (white).

In the following figures are shown: radius of gyration (R_g, top-left) ; number of salt bridges (SB, top-right); total solvent-accessible surface area (SASA, middle-left); hydrophobic SASA (hSASA) of the H-domain (middle-right); SASA of residues belonging to the F-domain (1-88, bottom-left); hSASA of the F-domain (bottom-right).

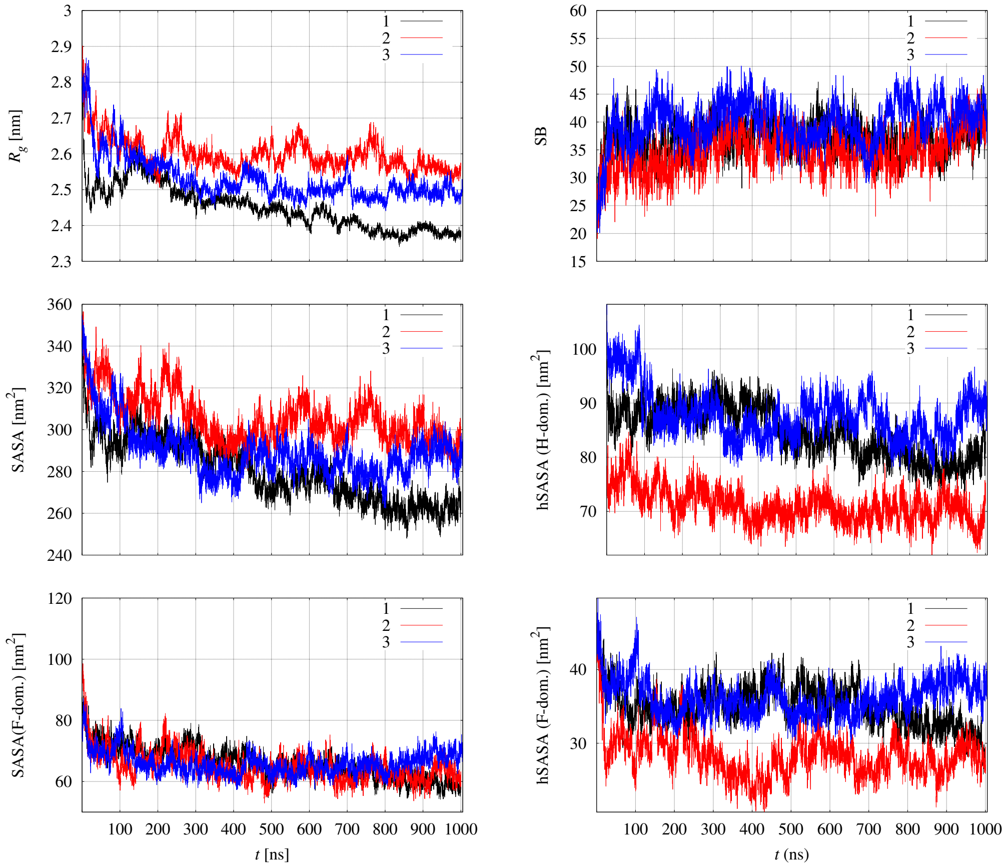

Figure S5. Top: Time evolution of structural parameters along with the whole trajectories **1** (black), **2** (red), and **3** (blue). Hydrophobic residues are Ala, Val, Leu, Ile, Pro, Met, and Trp. F-domain includes residues 1-88, H-domain 89-530.

As expected, all simulations show compaction of the protein. This occurs because AlphaFold and SwissModel predict extended configurations where available structural information is missing. Once the initially extended configurations are inserted into a model of the solution environment, the extended regions collapse into more rigid and

structured regions that change only slightly afterwards. However, the extent of this collapse significantly differs among the simulations. Simulation **1** shows a more compact initial configuration than **2** and **3**. At the end of the settling into the environment **1** achieves a final configuration that is more compatct compared to what one finds in simulations **2** and **3**, in terms of radius of gyration and SASA. This compaction is produced by the collapse of protein disordered regions towards the more structured H-domain. Disordered regions include the extended loops connecting different segments of the scaffold and the N-terminus, which is the candidate F-domain (residues 1-88). A significant contribution of the F-domain is visible from Figure S5, comparing middle and bottom left panels: the decrease of about 30 nm^2 of the F-domain SASA along trajectory **1** accounts for about 1/2 of the decrease of the total SASA ($\sim$70 nm^2). Being the F-domain formed by about 1/5 of the residues of the Hyd protein, this is a large relative contribution to protein compaction. Conversely, simulations **2** and **3** achieve a state where the total SASA is at least 20 nmˆ2 larger than **1**. This difference is due to the fact that the hydrophobic SASA is, in particular in simulation **2**, smaller than in **1** since the beginning (middle-left panel). The SwissModel construction starts from an H-domain built on the basis of a crystal structure of *Cr* Hyd (PDB 4R0V), thus showing a well-formed hydrophobic core characterized by a low exposure of hydrophobic residues. During the simulation **2** in water the hydrophobic cores of both H- and F-domains reduces the exposure of hydrophobic residues, while increasing the total exposure to the solvent.

The time-evolution of SB and hSASA values displayed in Figure S5 (right panels) shows that there is a stable network of electrostatic interactions within the protein. The network is strengthened in all simulations (top-right panel). The better hindering of hydrophobic residues in simulation **2** compared to **1** and, to a major extent, to **3** occurs keeping the network of electrostatic interactions almost unchanged.

The average RMSD values of the clusters along simulation **1** are, for $[2Fe]_H$, $[4Fe4S]_H$ and $[4Fe4S]_F$, 0.23±0.06, 0.07±0.01, 0.11±0.02 Å, respectively. In particular, it can be noticed an extended motion of H atom in the NH group of the *adt* ligand. This result holds for simulations **2** and **3**.

In the main text and in the following, we analyze in more details the differences among the three simulations as for the last 200 ns of trajetory, thus discarding the settling of initial configurations in the water environment.

To measure the mobility of residues along with the protein sequence we made the principal components analysis (PCA) (see Methods for details). The PCA of backbone heavy atoms was made in order to compare the collective motions of the protein backbone as described in the last 200 ns of simulations. In Figure S6 the root-mean square fluctuation (RMSF) computed projecting the trjactory of heavy backbone atoms on the first 4 eigenvectors of the covariance matrix are displayed. Only values for Cα atoms of each residue are displayed for clarity.

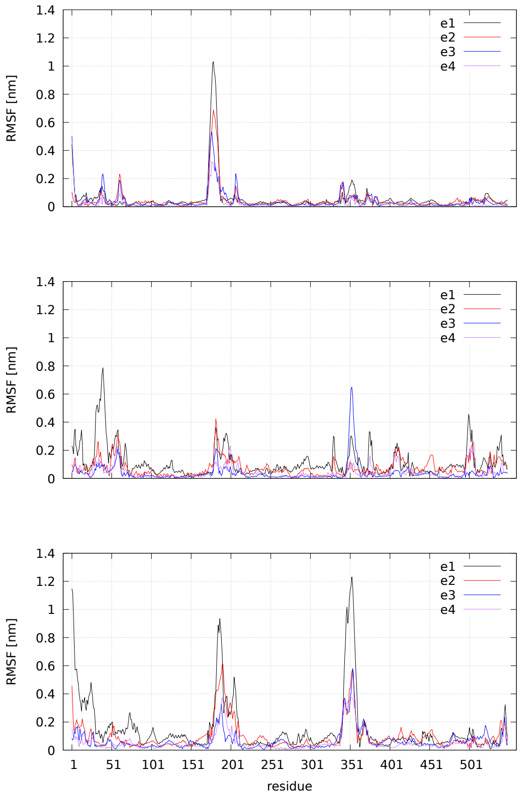

Figure S6. Root-mean square fluctuation of Cα atoms (RMSF) due to first 4 eigenvectors of covariance matrix (e1-4). Top: simulation **1**. Middle: simulation **2**. Bottom: simulation **3**.

The comparison between the simulations shows that simulation **1** ends, after 800 ns of simulation (before the final 200 ns used for analysis) towards a low fluctuating structure. Fluctuations are concentrated on the first few residues of the N-terminus and on the long loop connecting helices h3 and h4 (Arg 168-Cys 224). The latter loop encompasses a hydrophylic region and the largest fluctuation is around Ala 178, belonging to a 5-Ala segment (Ala 178-Ala 182) in the middle of the loop. Fluctuations are larger in simulation **3** than in **1**, showing a lower stability of the configuration reached after the first 800 ns of simulation **3** compared to that reached in the same time (800 ns) by simulation **1**. In particular the N-terminus is fluctuating even at Cys residues involved in the binding of the F-cluster (Table 1), with a significant stress around Fe atoms. The loop around residue 351 (h7-h8) is also fluctuating.

The difference of sampled space spanned by the simulations is confirmed by the PCA analysis: the normalized overlap of the covariance matrices [6] between **1** and **2** is 0.160 and with **3** is 0.144. The fluctuations in the last 200 ns of simulation **2** are as sparsed on the whole residues as in simulation **3**, but smaller in amplitude. The major difference compared to simulation **1** is in the behviour of the long h7-h8 loop. The reduction of fluctuations in this region increases the fluctuations in the N-terminus. The different behaviour of this loop and N-terminus in simulations **1** and **2** displays an important marker of mutual interactions between the H- and F-domains (see below).

As discussed in a recent work [7], active [FeFe] hydrogenases, with the exception of *Cr*, show that the $[4Fe4S]_H$-$[4Fe4S]_F$ distance (measured as the distance between the centers of mass) is in the range 11-14 Å. In our case, we have two models in which the distance between the two 4Fe4S clusters is in the range 12-20 Å (simulations **1** and **2**) and 25-40 Å (simulation **3**). In Figure S7 we report the time-evolution of the distance between the 4Fe4S centers of mass along the three simulations. The comparison shows that simulations **1** and **2** better agree with previous observations. The difference between the simulations is to be

Table S2. Average (over last 200 ns of simulations) of relative solvent accessible surface area (SASA) in the various FeS clusters.

Simulation	$[4Fe4S]_F$	$[4Fe4S]_H$	$[2Fe]_H$
**1**	0.07±0.02	0.01±0.01	0.06±0.02
**2**	0.03±0.03	0.03±0.02	0.06±0.02
**3**	0.05±0.03	0.03±0.02	0.05±0.02

ascribed to different model constructions because most of the change in distance is spanned during the first 300 ns of simulation.

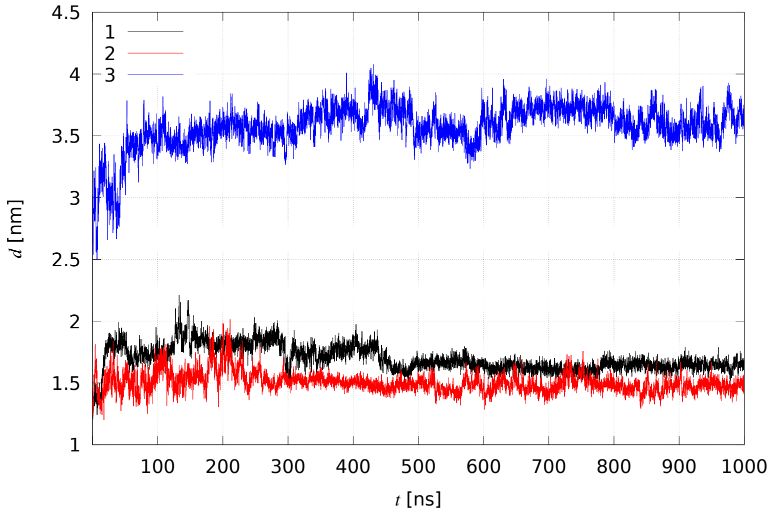

Figure S7. Time evolution of distance between 4Fe4S cluster centers of mass along 1 μs. Black: simulation **1**. Red: simulation **2**. Blue: simulation **3**.

In Table S2 we report the average of the relative SASA of different FeS clusters in the three simulations. Relative SASA is defined as the ratio between the measured SASA of the given group of atoms and the maximal SASA of the same group of atoms. The maximal SASA is computed deleting the protein matrix, therefore this quantity measures the fraction of SASA that is not buried by the protein matrix. With the exclusion of the distance between clusters (see Figure S7) SASA values are consistent with active hydrogenase, since all clusters display low accessibility to water (see also the discussion about Fe_d water accessibility). In simulation **3**, the F-domain partly unfolds, auxiliary cluster leaves its initial position, and the surface of the H-domain becomes more unprotected than in simulation **1**. In particular, the change of the F-domain is characterized by the breaking of the short N-terminal β-strand. On the other hand, the short helical motifs in the F-domain appear stable in simulation **3**, thus hindering the approach of $[4Fe4S]_F$ towards the surface of the H-domain. The compaction of a slightly folded F-domain in simulation **2** hinders water accessibility to the accessory cluster, but with no measurable effect on the accessibility of other clusters. However, the change of water accessibility to Fe_d as measured by the positions of explicit water molecules (see Figure 5 in main text) is affected by the different arrangement of the F-domain in simulations **3** and **1**.

In Table S3 we display the RMSD of the structured parts in the H-domain with respect to crystal structures of active Hyd forms (thus containing the H-cluster in one of the reduced forms). The final deviations are consequence of the settling of the initial organization chosen for each simulation. Those simulations (**1** and **3**) started from the H-domain derived by AlphaFold display low values for both the 11-helix bundle and the 2 β-sheets, including the reference structure of *Cr*. Simulation **2**, started from an oxidized form of HydA1 in

Table S3. RMSD (Å) of heavy backbone atoms (N, Cα, C, O). As in Table 2 (main text) the left value is for the whole H-domain scaffold, middle is for the 11 helix-bundle, right is for the 7 β-strands. The final configuration (1 μs) is used to represent each simulation. Reference crystal structures are the same used in Table 2 in main text. When one simulation is compared to itself, the reference configuration is the initial one.

reference/target	**1**	**2**	**3**
CpI	3.3/3.7/1.7	6.7/4.3/9.2	3.8/4.1/2.0
Dd Hyd	3.0/3.3/1.7	6.5/3.9/9.2	3.4/3.7/1.9
Cr Hyd	3.6/4.1/1.3	6.4/3.3/9.1	4.9/5.5/1.7
**1**	3.0/3.4/1.3	5.8/3.1/8.8	3.3/3.6/1.7
**2**		3.1/3.1/2.7	6.7/4.1/9.3
**3**			2.7/2.7/2.2

Cr (PDB 4R0V [8]), is different from the available mature crystal structures mainly in the organization of the 7 β strands. During each simulation the H-domain scaffold does not change significantly, since RMSD from each inital configuration is always smaller than 4 Å. The relaxation of the disordered loops present in all simulations (not included in the calculation of RMSD data of Tables 2 and S3) affects more significantly the interactions betwee H- and F-domains than the interactions between the structural elements within the H-domain.

In agreement with the observations about SASA of different domains and sub-domains (see below and Figure S5 with related discussion), RMSD values indicate that the structural change of the initial structures occur, in simulations **1** and **3**, keeping the protein scaffold almost rigid, while N-terminal (F-domain) and loops contribute for the largest part to structural relaxation. However, interestingly, the whole protein backbone and the H-domain scaffold change more significantly when the F-domain is more structured. This occurs in simulation **3** since the beginning and in simulation **2** during the simulation (see below).

4. Representative structures

The structures displayed in Figure 7 in the main text are provided in the PDB folder of SM: last_1.pdb, last_2.pdb, and last_3.pdb for simulations **1**, **2**, and **3**, respectively. Those are configurations obtained at the end of the 1 μs simulations.

1. Kabsch, W.; Sander, C. Dictionary of protein secondary structure - Pattern-recognition of hydrogen-bonded and geometrical features. *Biopolymers* **1983**, *22*, 2577–2637. doi:10.1002/bip.360221211.
2. Chang, C.H.; Kim, K. Density Functional Theory Calculation of Bonding and Charge Parameters for Molecular Dynamics Studies on [FeFe] Hydrogenases. *J. Chem. Theory Comput.* **2009**, *5*, 1137–1145. doi:10.1021/ct800342w.
3. Humphrey, W.; Dalke, A.; Schulten, K. VMD visual molecular dynamics. *J. Molec. Graphics* **1996**, *14*, 33–38. http://www.ks.uiuc.edu/Research/vmd, doi:10.1016/0263-7855(96)00018-5.
4. Phillips, J.C.; Hardy, D.J.; Maia, J.D.C.; Stone, J.E.; Ribeiro, J.V.; Bernardi, R.C.; Buch, R.; Fiorin, G.; Hénin, J.; Jiang, W.; McGreevy, R.; Melo, M.C.R.; Radak, B.K.; Skeel, R.D.; Singharoy, A.; Wang, Y.; Roux, B.; Aksimentiev, A.; Luthey-Schulten, Z.; Kalé, L.V.; Schulten, K.; Chipot, C.; Tajkhorshid, E. Scalable molecular dynamics on CPU and GPU architectures with NAMD. *J. Chem. Phys.* **2020**, *153*, 044130. http://www.ks.uiuc.edu/Research/namd, doi:10.1063/5.0014475.
5. Vermaas, J.V.; Hardy, D.J.; Stone, J.E.; Tajkhorshid, E.; Kohlmeyer, A. TopoGromacs: Automated Topology Conversion from CHARMM to GROMACS within VMD. *J. Chem. Inform. Model.* **2016**, *56*, 1112–1116. doi:10.1021/acs.jcim.6b00103.

6. Hess, B. Convergence of sampling in protein simulations. *Phys. Rev. E* **2002**, *65*, 031910–10. doi:10.1103/PhysRevE.65.031910.
7. Puthenkalathil, R.C.; Ensing, B. Fast Proton Transport in FeFe Hydrogenase via a Flexible Channel and a Proton Hole Mechanism. *J. Phys. Chem. B* **2022**, *126*, 403–411. doi:10.1021/acs.jpcb.1c08124.
8. Swanson, K.D.; Ratzloff, M.W.; Mulder, D.W.; Artz, J.H.; Ghose, S.; Hoffman, A.; White, S.; Zadvornyy, O.A.; Broderick, J.B.; Bothner, B.; King, P.W.; Peters, J.W. [FeFe]-Hydrogenase Oxygen Inactivation Is Initiated at the H Cluster 2Fe Subcluster. *J. Am. Chem. Soc.* **2015**, *137*, 1809–1816. doi:10.1021/ja510169s.
