# Supplementary figures and images for: Predicting the Structure of Enzymes with Metal Cofactors: The Example of [FeFe] Hydrogenases
Source: Int J Mol Sci. 2024 Mar 25;25(7):3663. doi: 10.3390/ijms25073663 (PMC11011570; doi:10.3390/ijms25073663)

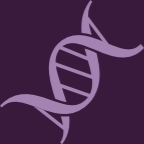

# International Journal of *Molecular Sciences*

Supplement: Supplementary file 1 [file ijms-25-03663-s001.zip › ijms-2857370-SM/SM-tex/Definitions/ijms-logo-eps-converted-to.pdf]

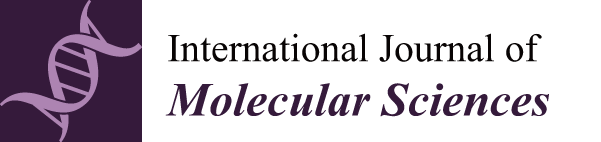

Supplement: Supplementary file 1 [file ijms-25-03663-s001.zip › ijms-2857370-SM/SM-tex/Definitions/ijms-logo.png]

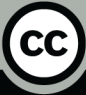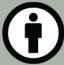

BY

Supplement: Supplementary file 1 [file ijms-25-03663-s001.zip › ijms-2857370-SM/SM-tex/Definitions/logo-ccby-eps-converted-to.pdf]

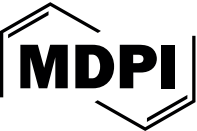

Supplement: Supplementary file 1 [file ijms-25-03663-s001.zip › ijms-2857370-SM/SM-tex/Definitions/logo-mdpi-eps-converted-to.pdf]

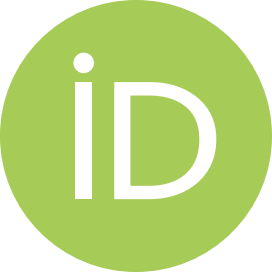

Supplement: Supplementary file 1 [file ijms-25-03663-s001.zip › ijms-2857370-SM/SM-tex/Definitions/logo-orcid.pdf]

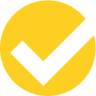

check for  
updates

Supplement: Supplementary file 1 [file ijms-25-03663-s001.zip › ijms-2857370-SM/SM-tex/Definitions/logo-updates-eps-converted-to.pdf]

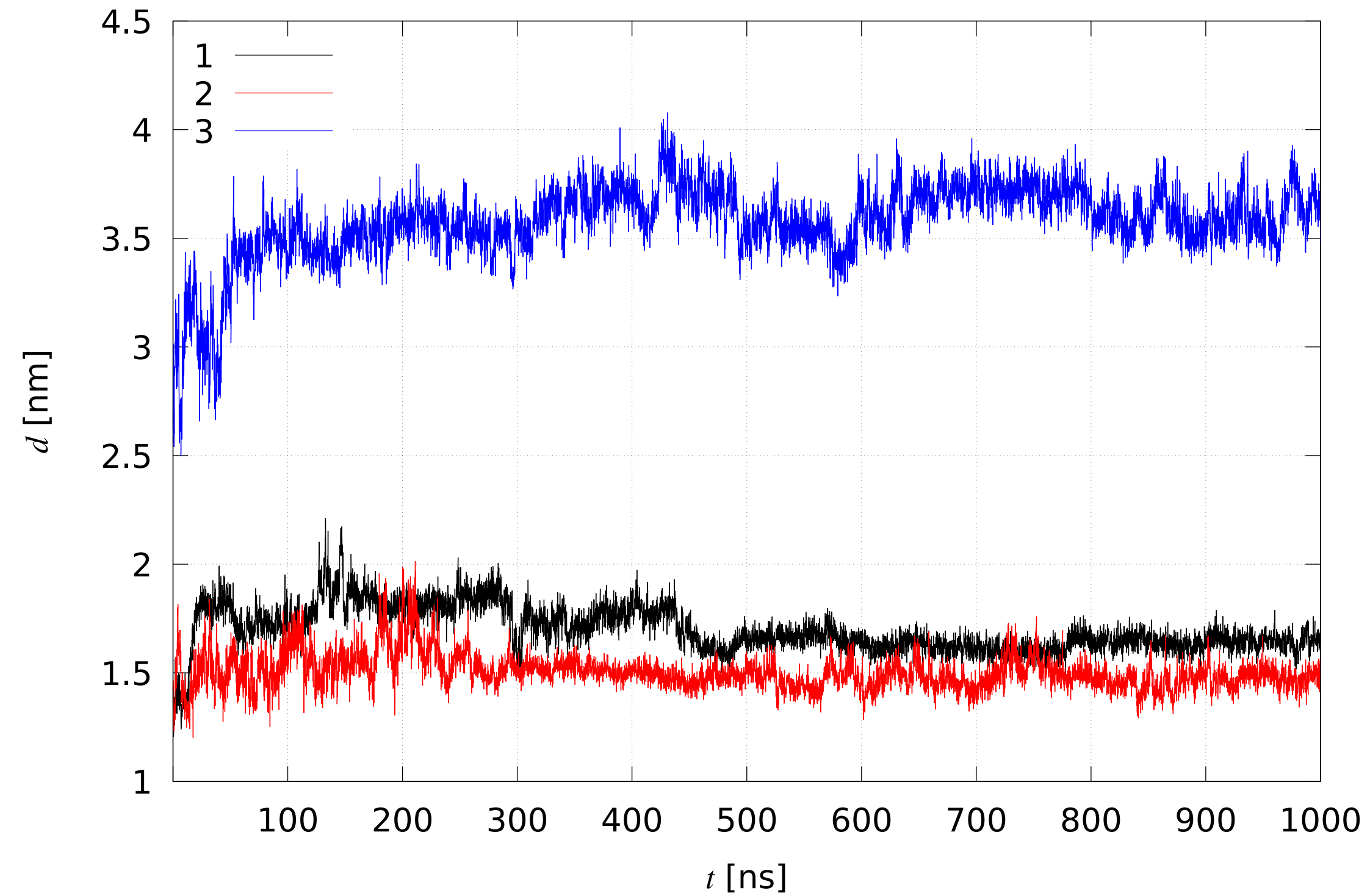

Supplement: Supplementary file 1 [file ijms-25-03663-s001.zip › ijms-2857370-SM/SM-tex/figures/clustd.pdf]

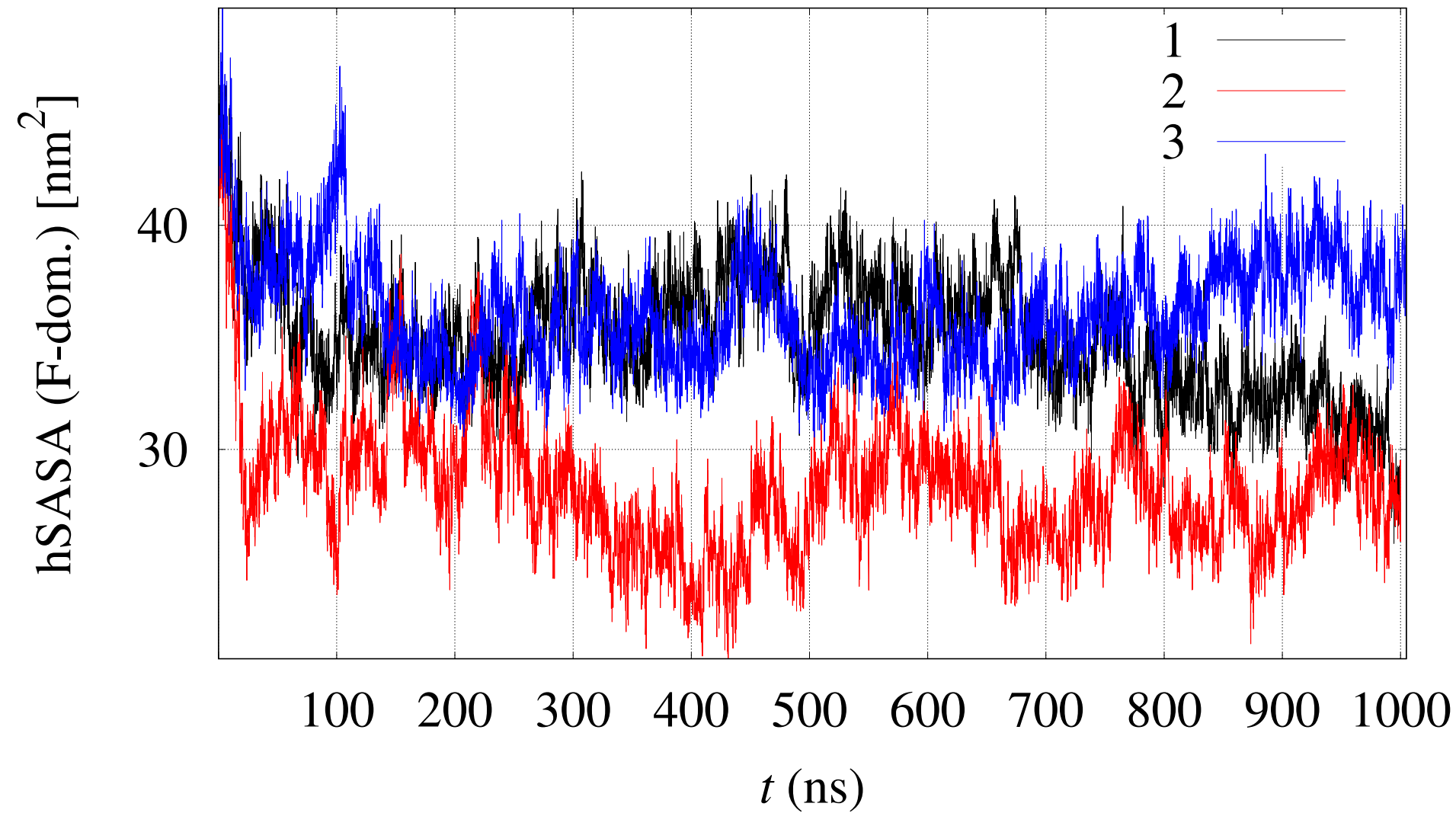

Supplement: Supplementary file 1 [file ijms-25-03663-s001.zip › ijms-2857370-SM/SM-tex/figures/hSASAF.pdf]

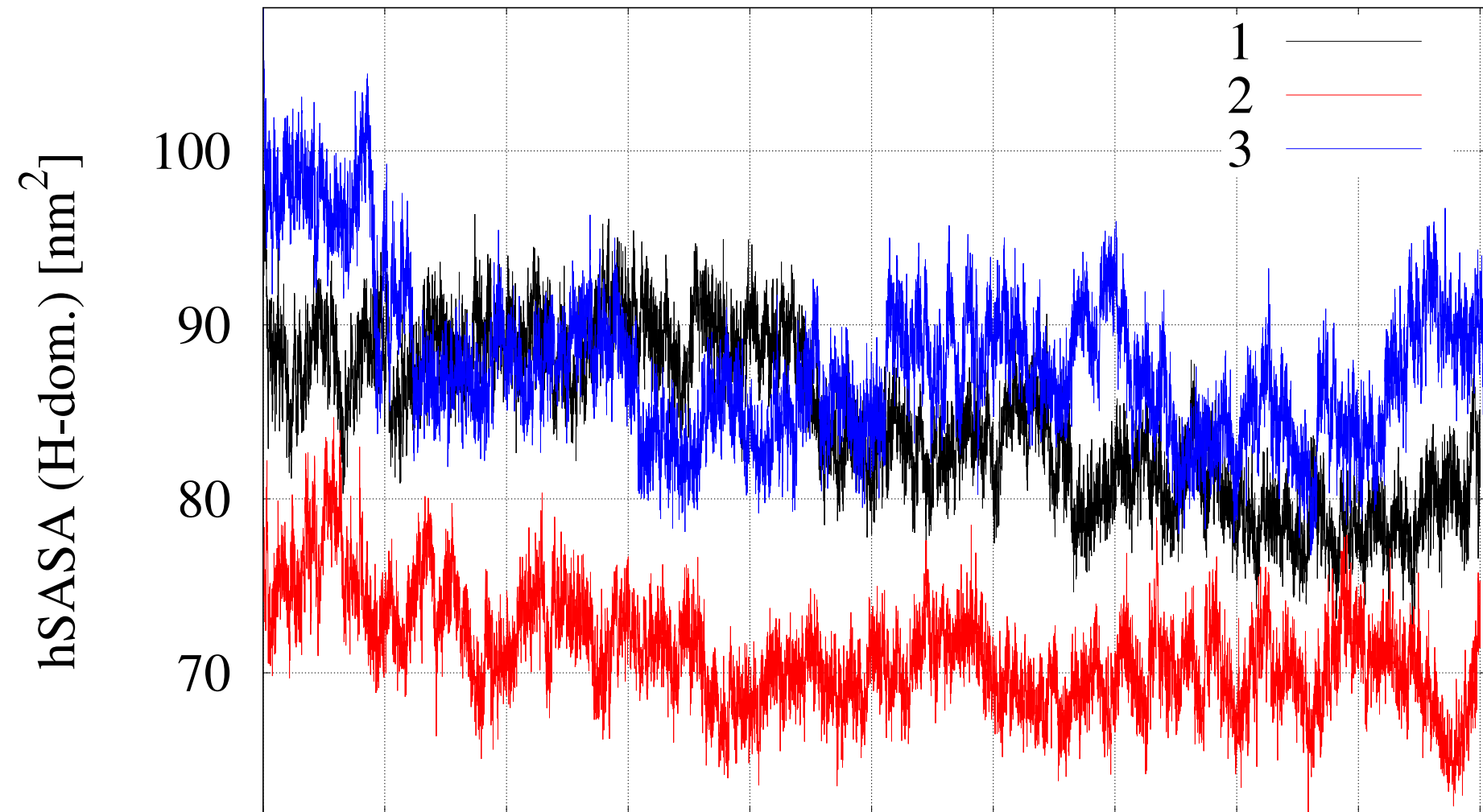

Supplement: Supplementary file 1 [file ijms-25-03663-s001.zip › ijms-2857370-SM/SM-tex/figures/hSASAH.pdf]

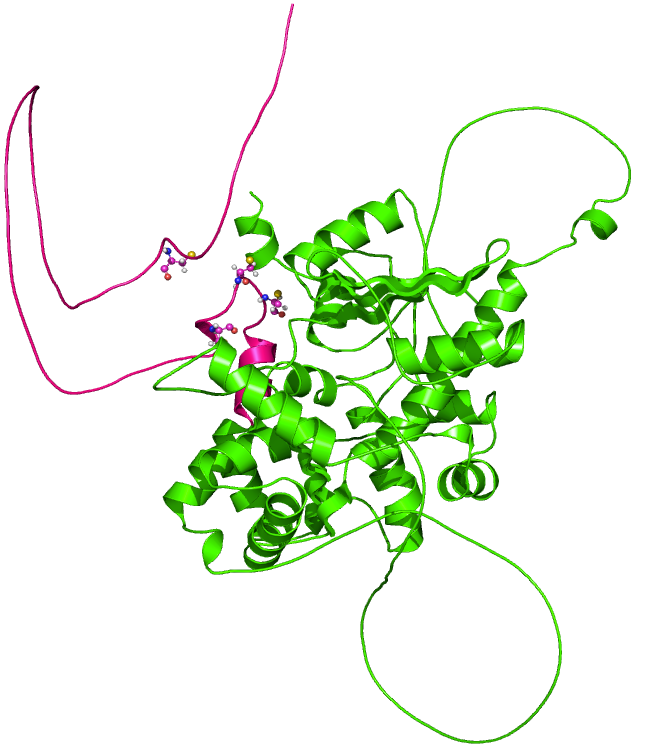

Supplement: Supplementary file 1 [file ijms-25-03663-s001.zip › ijms-2857370-SM/SM-tex/figures/in1.png]

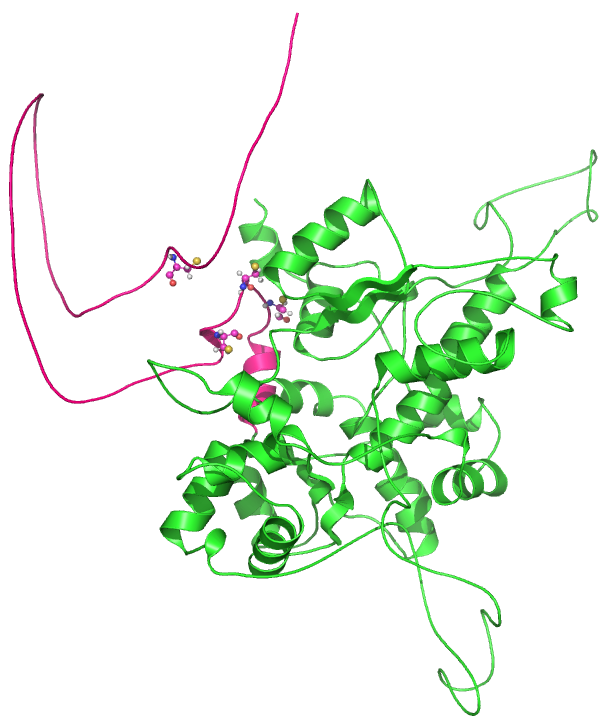

Supplement: Supplementary file 1 [file ijms-25-03663-s001.zip › ijms-2857370-SM/SM-tex/figures/in2.png]

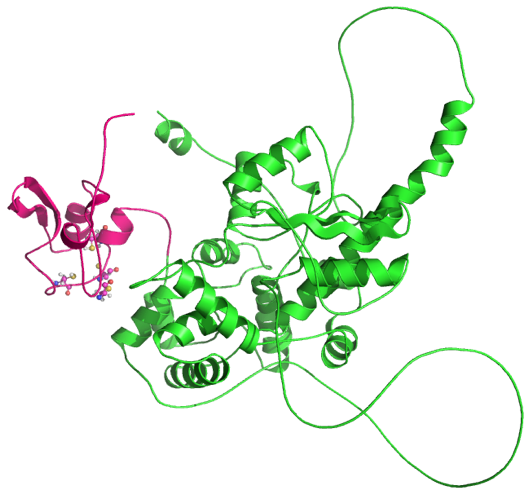

Supplement: Supplementary file 1 [file ijms-25-03663-s001.zip › ijms-2857370-SM/SM-tex/figures/in3.png]

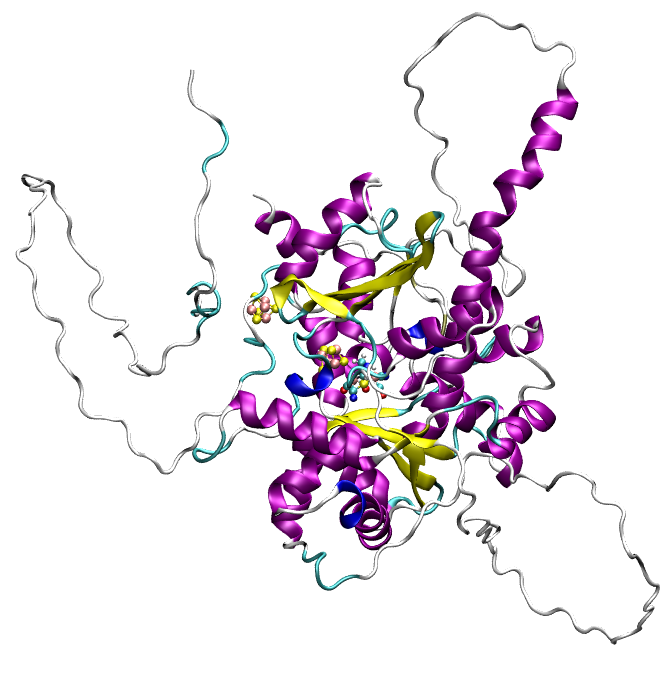

Supplement: Supplementary file 1 [file ijms-25-03663-s001.zip › ijms-2857370-SM/SM-tex/figures/min1.png]

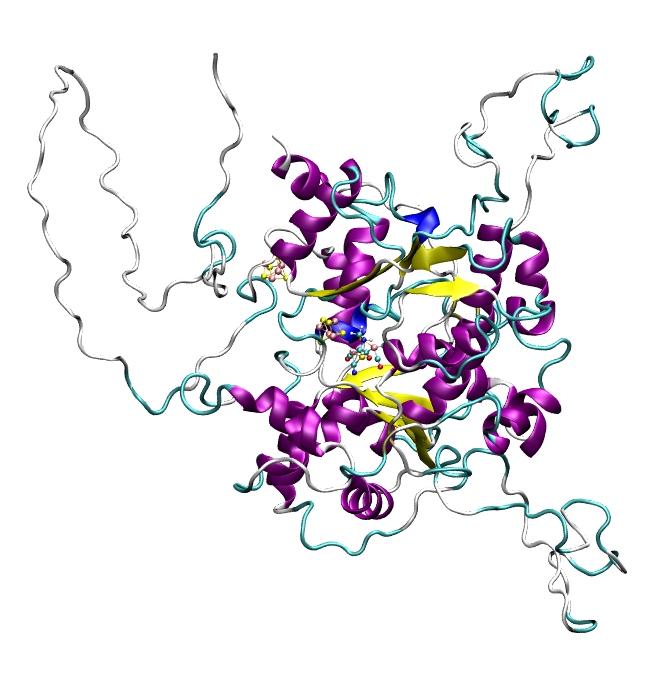

Supplement: Supplementary file 1 [file ijms-25-03663-s001.zip › ijms-2857370-SM/SM-tex/figures/min2.png]

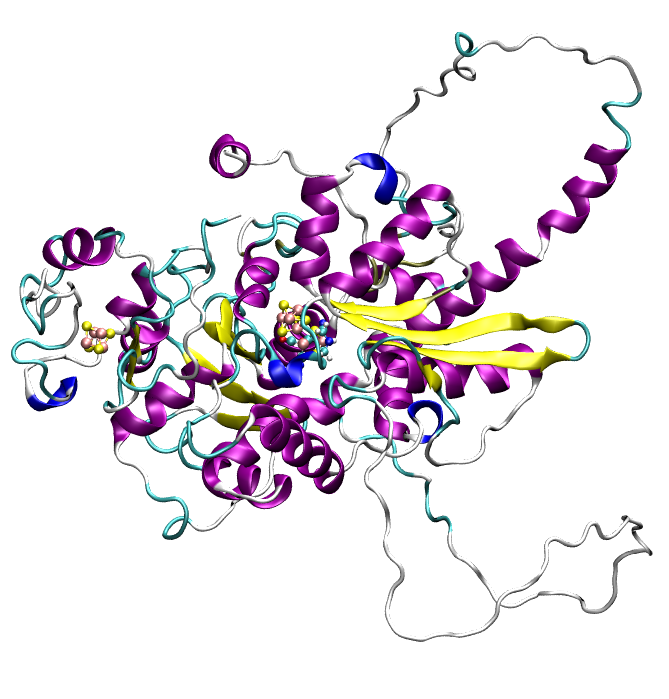

Supplement: Supplementary file 1 [file ijms-25-03663-s001.zip › ijms-2857370-SM/SM-tex/figures/min3.png]

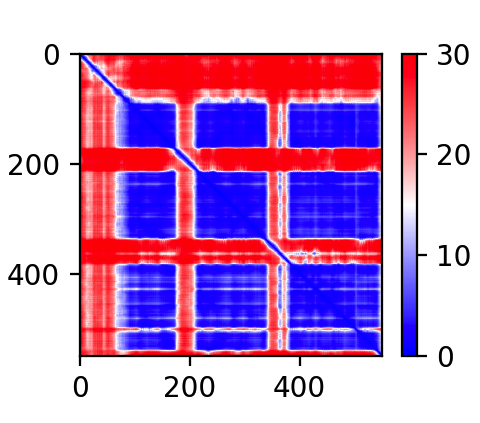

Supplement: Supplementary file 1 [file ijms-25-03663-s001.zip › ijms-2857370-SM/SM-tex/figures/PAE.png]

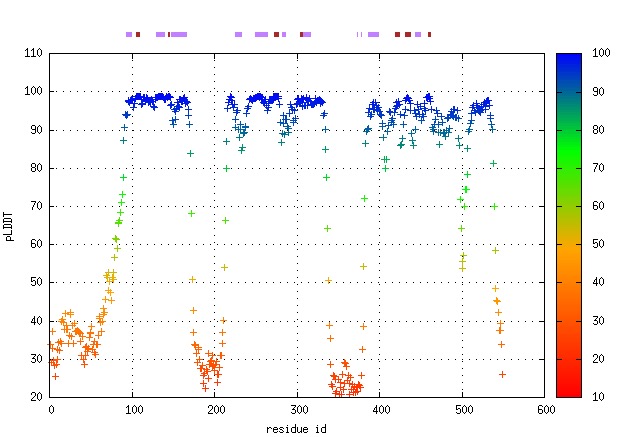

Supplement: Supplementary file 1 [file ijms-25-03663-s001.zip › ijms-2857370-SM/SM-tex/figures/plDDT.png]

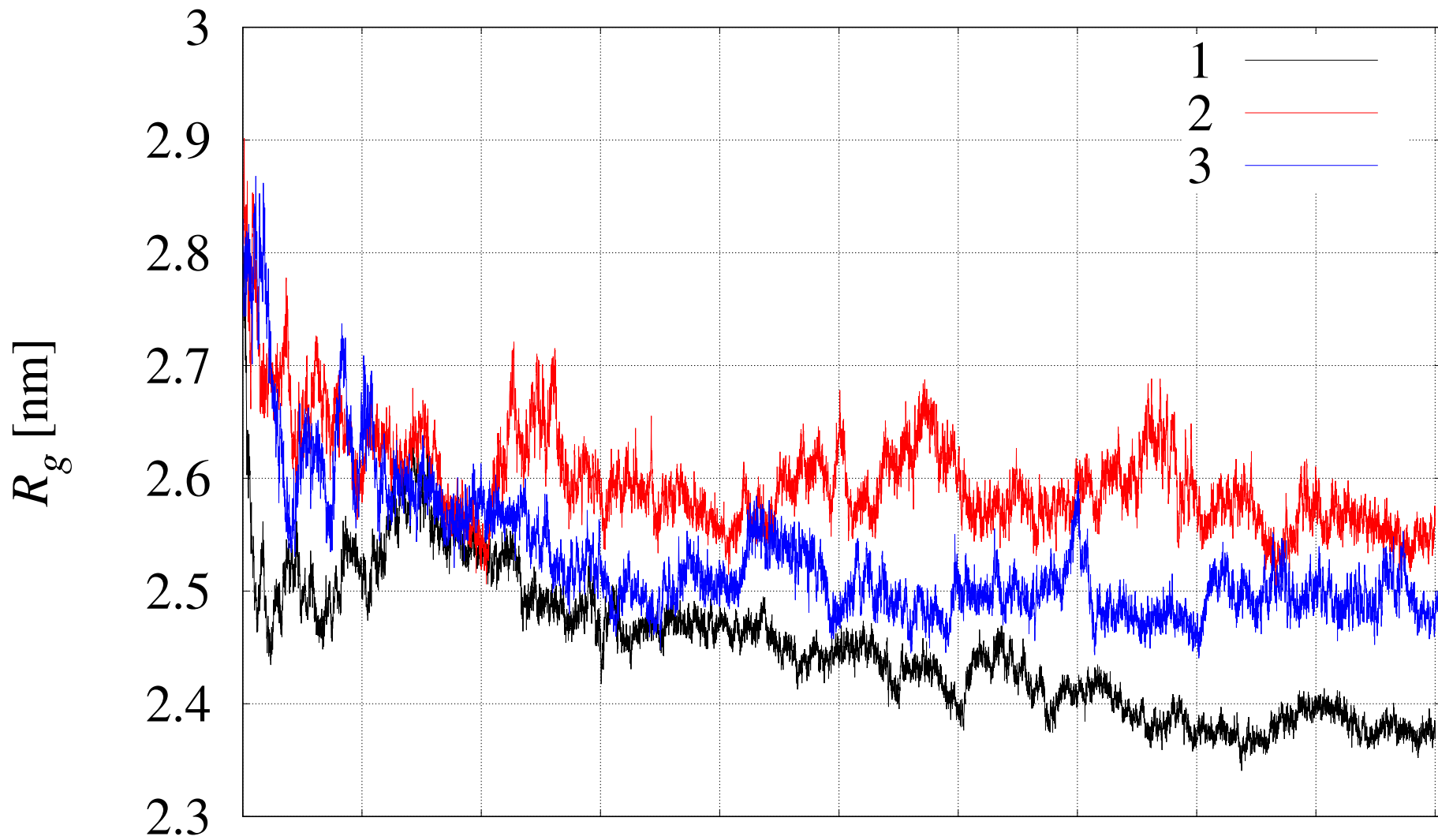

Supplement: Supplementary file 1 [file ijms-25-03663-s001.zip › ijms-2857370-SM/SM-tex/figures/Rg.pdf]

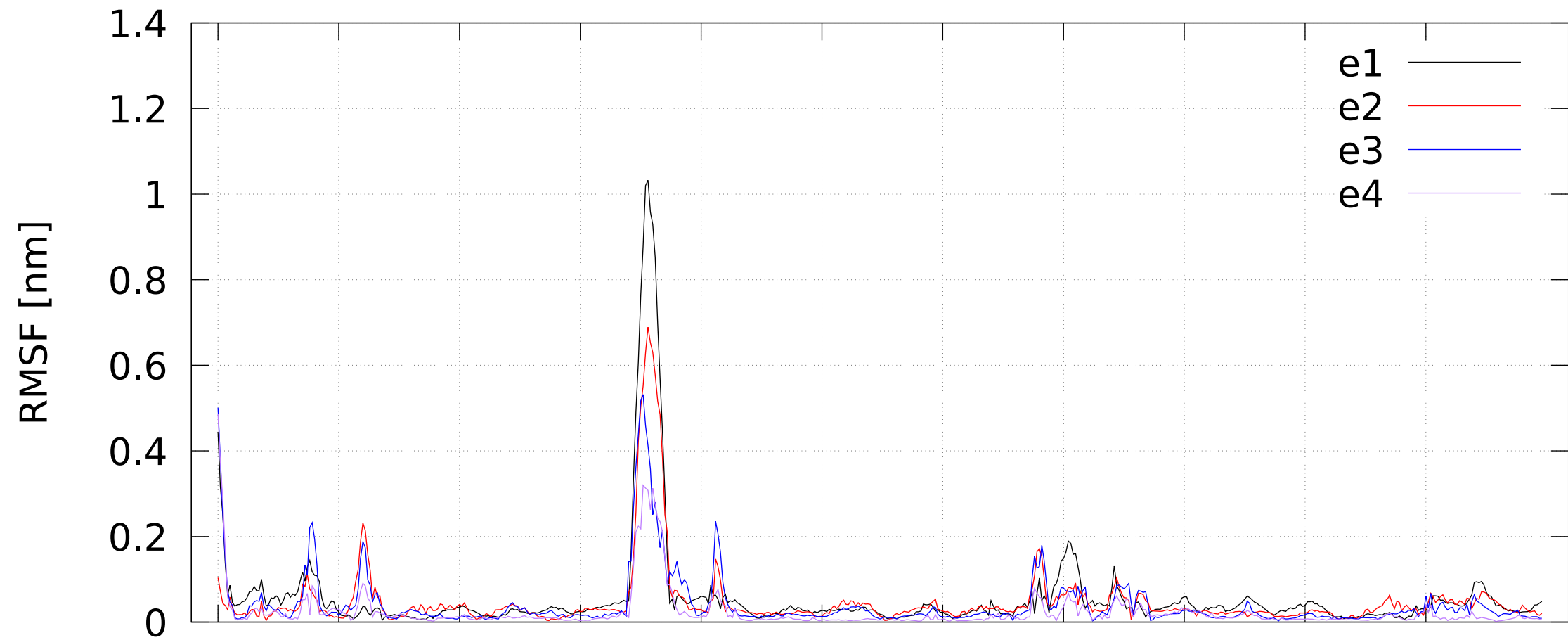

Supplement: Supplementary file 1 [file ijms-25-03663-s001.zip › ijms-2857370-SM/SM-tex/figures/RMSF1.pdf]

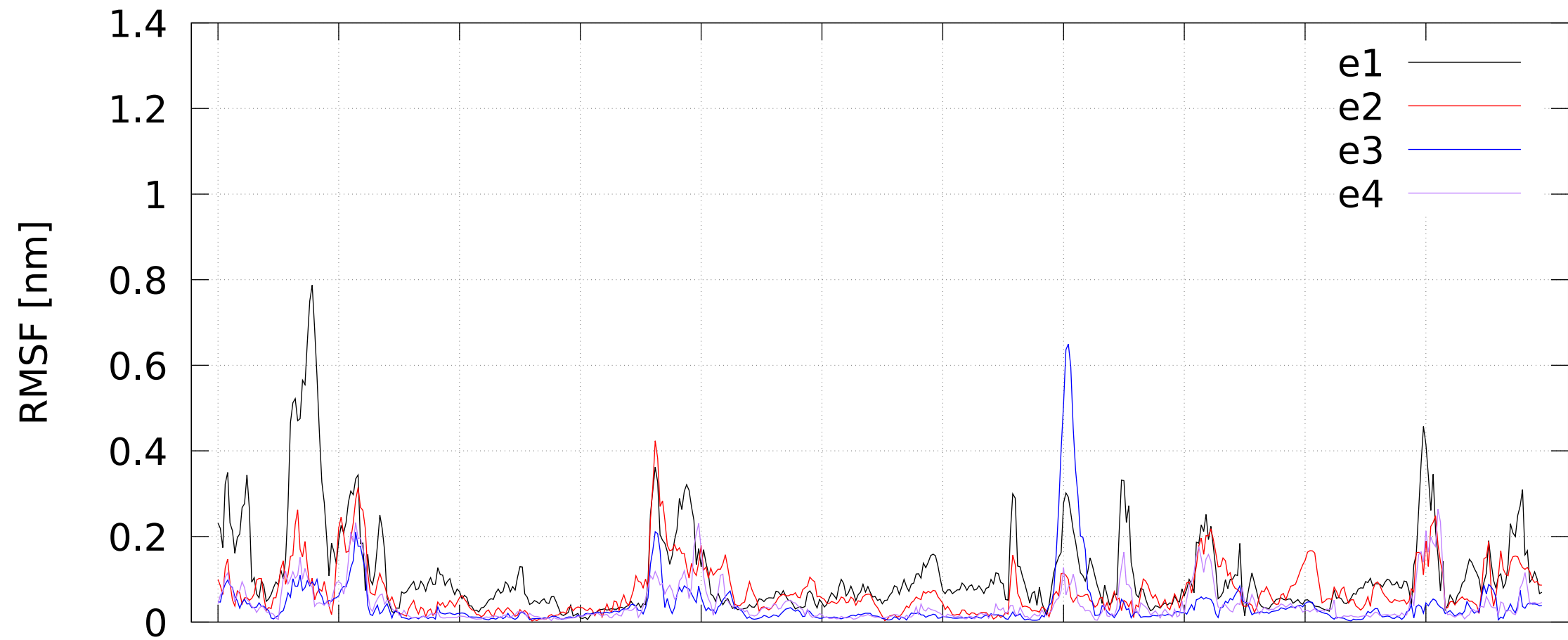

Supplement: Supplementary file 1 [file ijms-25-03663-s001.zip › ijms-2857370-SM/SM-tex/figures/RMSF2.pdf]

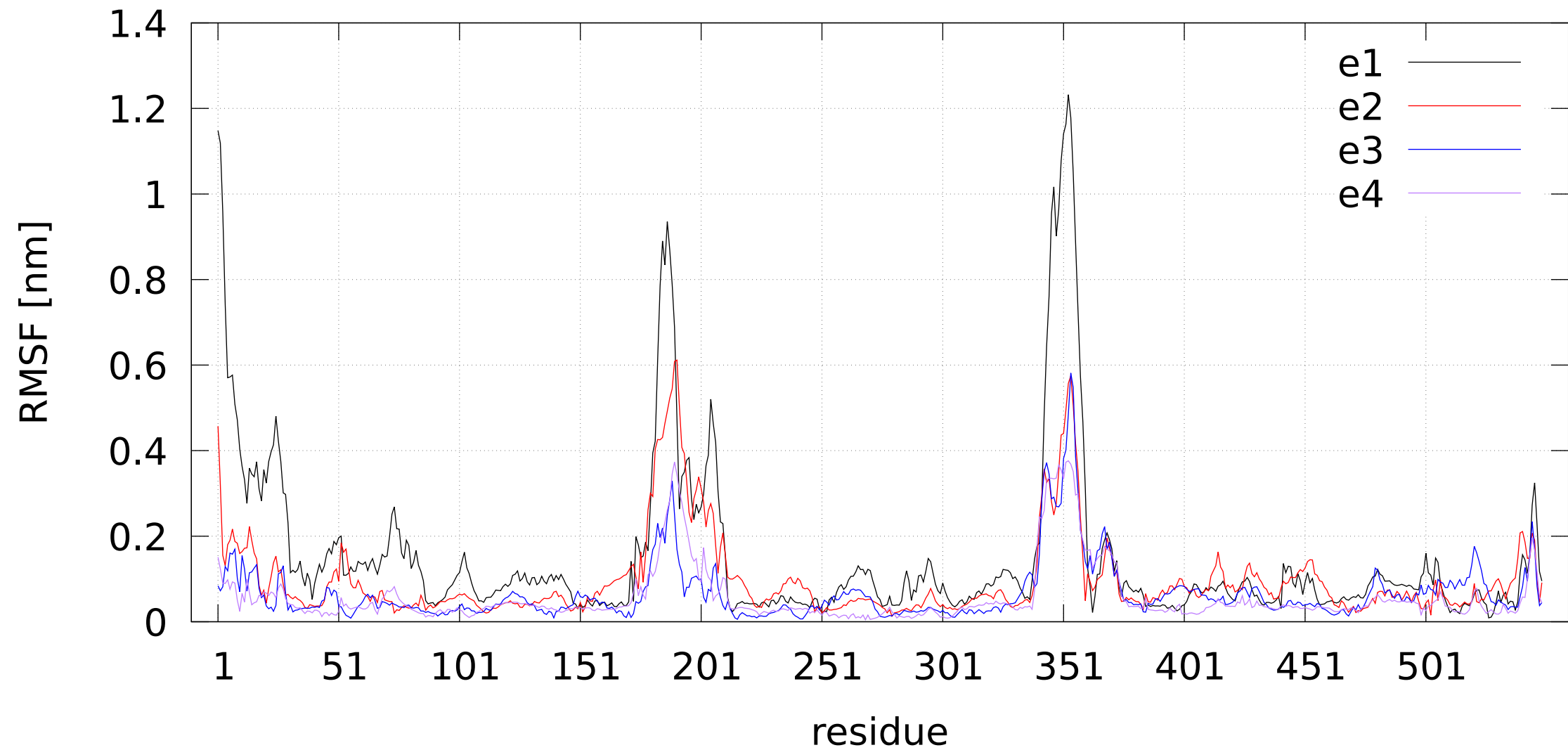

Supplement: Supplementary file 1 [file ijms-25-03663-s001.zip › ijms-2857370-SM/SM-tex/figures/RMSF3.pdf]

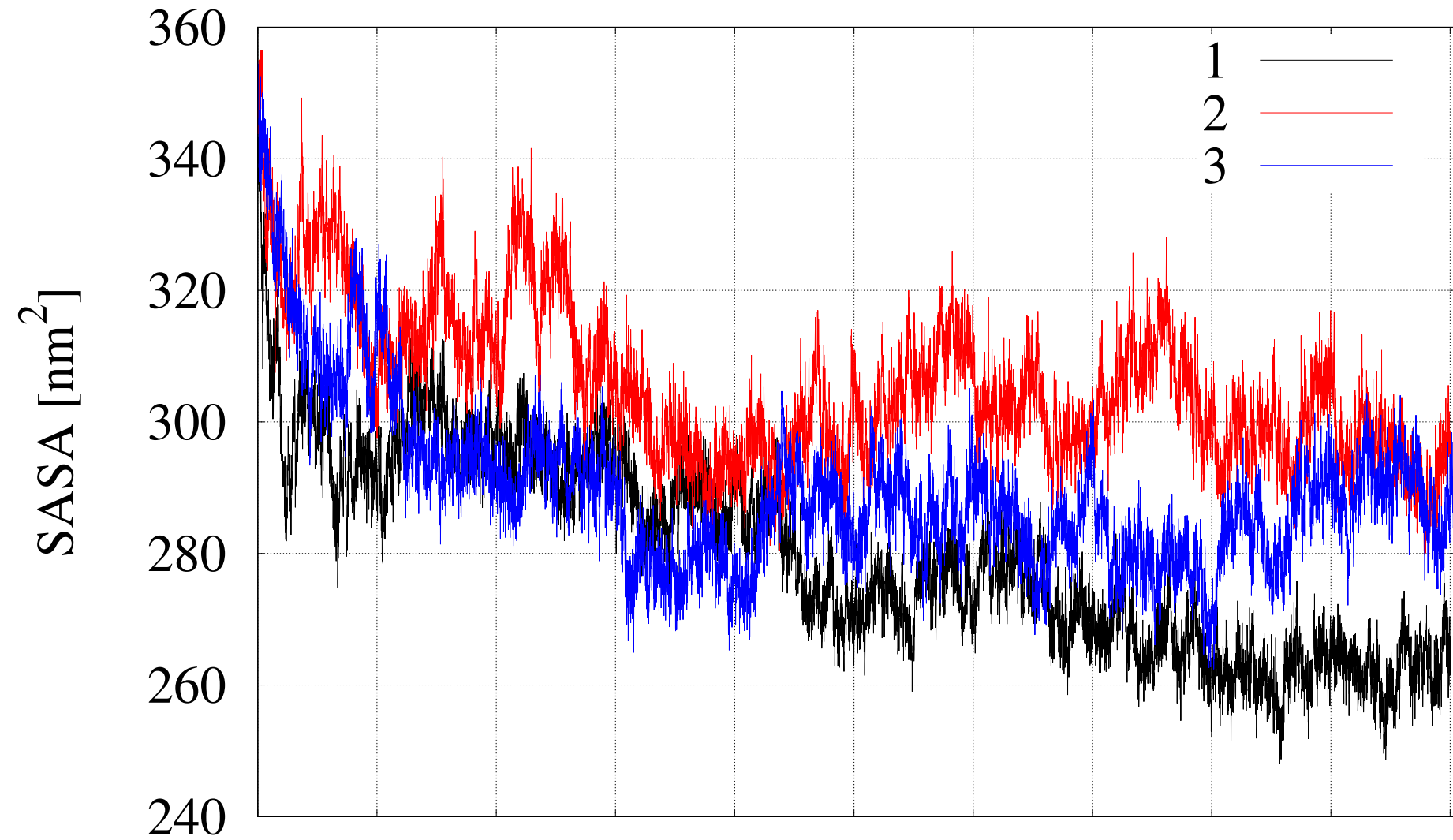

Supplement: Supplementary file 1 [file ijms-25-03663-s001.zip › ijms-2857370-SM/SM-tex/figures/SASA.pdf]

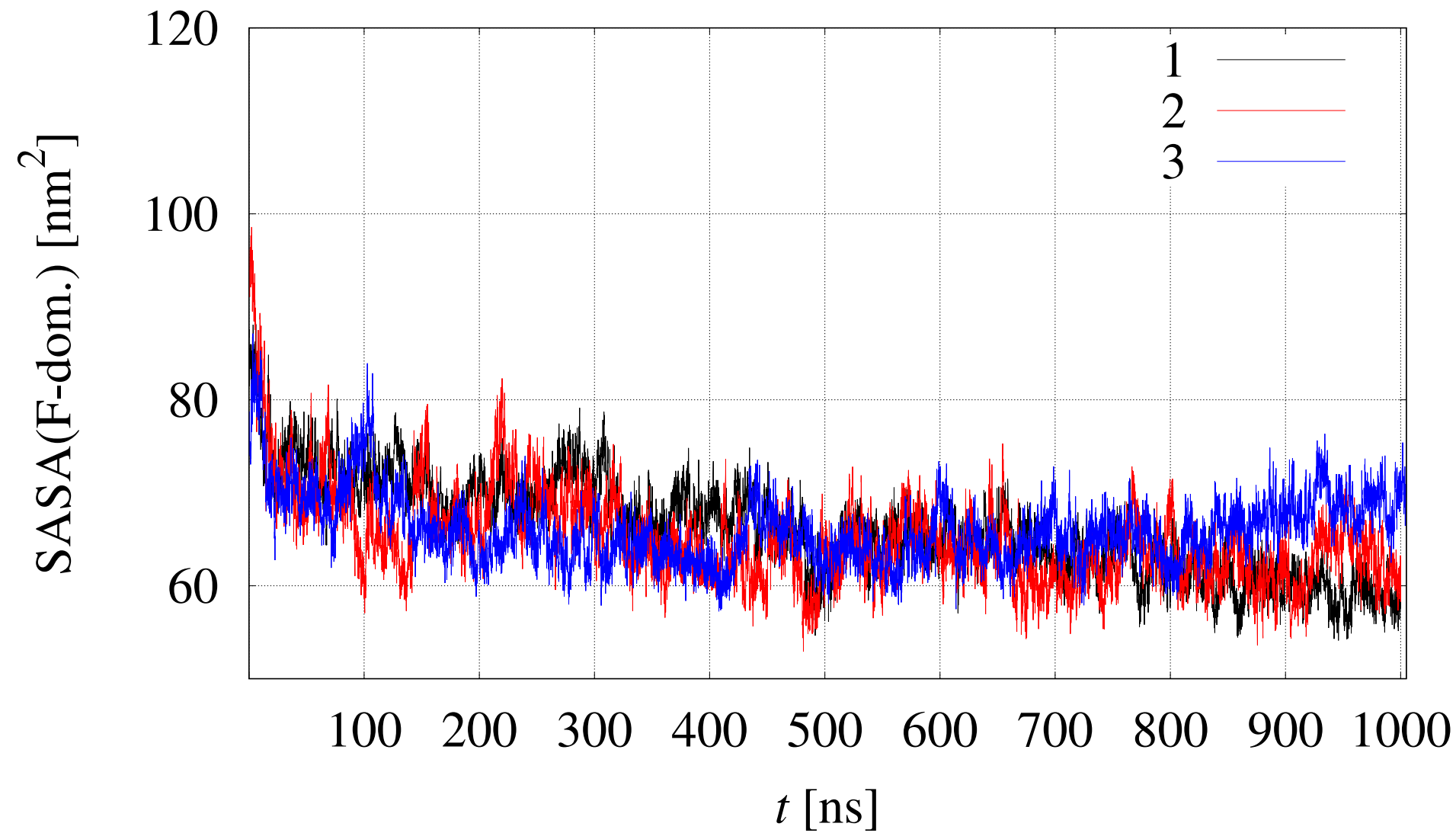

Supplement: Supplementary file 1 [file ijms-25-03663-s001.zip › ijms-2857370-SM/SM-tex/figures/SASAF.pdf]

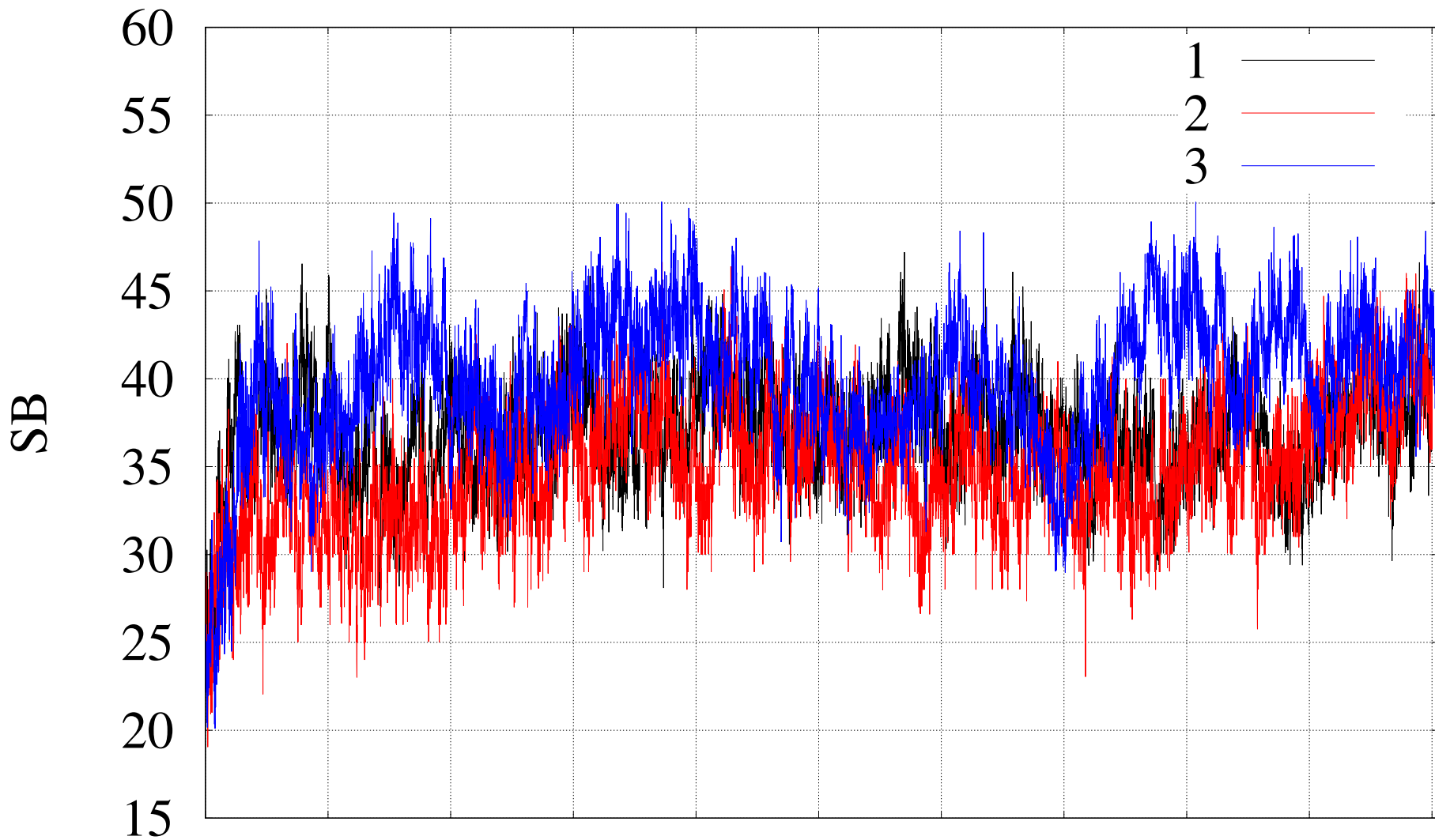

Supplement: Supplementary file 1 [file ijms-25-03663-s001.zip › ijms-2857370-SM/SM-tex/figures/SB.pdf]
